# Efficient Second Strand Cleavage during Holliday Junction Resolution by RuvC Requires Both Increased Junction Flexibility and an Exposed 5′ Phosphate

**DOI:** 10.1371/journal.pone.0005347

**Published:** 2009-04-28

**Authors:** Fekret Osman, Louise Gaskell, Matthew C. Whitby

**Affiliations:** Department of Biochemistry, University of Oxford, Oxford, United Kingdom; University of Minnesota, United States of America

## Abstract

**Background:**

Holliday junction (HJ) resolution is a critical step during homologous recombination. In *Escherichia coli* this job is performed by a member of the RNase H/Integrase superfamily called RuvC, whereas in *Schizosaccharomyces pombe* it has been attributed to the XPF family member Mus81-Eme1. HJ resolution is achieved through the sequential cleavage of two strands of like polarity at or close to the junction crossover point. RuvC functions as a dimer, whereas Mus81-Eme1 is thought to function as a dimer of heterodimers. However, in both cases the multimer contains two catalytic sites, which act independently and sequentially during the resolution reaction. To ensure that both strands are cleaved before the nuclease dissociates from the junction, the rate of second strand cleavage is greatly enhanced compared to that of the first. The enhancement of second strand cleavage has been attributed to the increased flexibility of the nicked HJ, which would facilitate rapid engagement of the second active site and scissile bond. Here we have investigated whether other properties of the nicked HJ are important for enhancing second strand cleavage.

**Principal Findings:**

A comparison of the efficiency of cleavage of nicked HJs with and without a 5′ phosphate at the nick site shows that a 5′ phosphate is required for most of the enhancement of second strand cleavage by RuvC. In contrast Mus81-Eme1 cleaves nicked HJs with and without a 5′ phosphate with equal efficiency, albeit there are differences in cleavage site selection.

**Conclusions:**

Our data show that efficient HJ resolution by RuvC depends on the 5′ phosphate revealed by incision of the first strand. This is a hitherto unappreciated factor in promoting accelerated second strand cleavage. However, a 5′ phosphate is not a universal requirement since efficient cleavage by Mus81-Eme1 appears to depend solely on the increased junction flexibility that is developed by the first incision.

## Introduction

Four-way DNA junctions (e.g. Holliday junctions (HJs), reversed replication forks, and displacement loops (D-loops)) are key intermediates in genetic recombination and perturbed DNA replication. They are normally formed between homologous chromosomes or sister chromatids, and consequently their timely processing is a prerequisite for successful chromosome segregation during cell division. An assortment of nucleases, helicases and topoisomerases process four-way DNA junctions. Amongst these are the HJ resolvases [1]–[3]. HJ resolvases are typically small homodimeric endonucleases that bind with structure-specificity to the HJ and introduce a pair of symmetrically placed incisions in strands of like polarity at or close to the junction crossover point. This type of dual incision resolves the HJ into two nicked duplexes, with each nick containing a 5′ phosphate and 3′ hydroxyl making them directly repairable by DNA ligase.

In addition to structure-specific binding, some HJ resolvases also exhibit sequence-specific DNA cleavage. An example of this type is RuvC from *Escherichia coli*, which cleaves nucleotide sequences with a 5′-^A^/_T_TT↓^G^/_C_-3′ consensus [4]. Such sequence specificity endows RuvC with an added level of substrate selectivity since efficient cutting is only achieved if both active sites within the homodimer are correctly positioned next to a strand with the right nucleotide sequence. HJs fulfil this requirement because they consist of two pairs of identical strands, and can undergo branch migration to relocate to sequences that are cleavable.

Proper resolution of a HJ requires that the dual incisions are made with perfect symmetry. However, the two active sites within a RuvC homodimer operate independently with respect to cleavage [5]. In principle this could be problematic if the junction branch migrated following the first incision and before the second incision is made, since widely spaced nicks would not result in junction resolution. Resolvases, like RuvC, avoid this by an acceleration of the second strand cleavage compared to the first, so that two incisions are made within the lifetime of a single binding event [1]. In the case of RuvC a 150-fold acceleration has been calculated [6]. The mechanism underlying this acceleration is thought to be due to the increase in junction flexibility caused by the first incision, which promotes interaction between the second active site and the scissile bond [1].

In addition to the HJ resolvases members of the XPF family of endonucleases have been implicated in processing four-way DNA junctions. Most notable in this grouping is Mus81, which functions with a partner protein called Eme1 (or Mms4 in *Saccharomyces cerevisiae*) [7]–[11]. This enzyme is conserved from yeasts to mammals, and, depending on the species, promotes the processing of stalled and/or broken replication forks, the repair of interstrand crosslinks, and the formation of crossover recombinants during meiosis [12], [13]. Like the HJ resolvases, Mus81-Eme1 can cleave fully ligated HJs, albeit the cut sites are asymmetrically-related and therefore the cleavage products cannot be directly repaired by DNA ligase [7], [14]–[16]. Moreover, it has a strong preference for binding and cleaving nicked HJs [14], [15], [17], and as such it is thought that these junctions represent its favoured substrate *in vivo* [12], [15], [17]. Similar to the enhancement of second strand cleavage by RuvC, efficient cleavage of nicked junctions by Mus81-Eme1 has been attributed to the increased flexibility of nicked HJs over fully ligated HJs, which enables the junction arm on the 5′ side of the nick to interact with a patch of basic residues near the active site [18].

Although nicked HJs are key intermediates/substrates of both RuvC and Mus81-Eme1 cleavage reactions it is not known whether the terminal chemistry at the nick site plays any role in the cleavage mechanism. In other words is the flexibility of a nicked HJ sufficient to promote efficient cleavage or are there other properties of nicked HJs that are needed? Here we have investigated whether the presence of a 5′ phosphate at the nick site affects either RuvC's or Mus81-Eme1's cleavage of a nicked HJ. In the case of Mus81-Eme1 the presence of a 5′ phosphate makes no difference to cleavage efficiency, suggesting that the flexibility of a nicked junction is the main factor in promoting its efficient cleavage. In contrast, a 5′ phosphate dramatically stimulates RuvC's cleavage of a nicked HJ. This suggests that junction flexibility on its own is insufficient to promote optimal second strand cleavage during HJ resolution by RuvC.

## Results

### A 5′ phosphate is needed for optimal cleavage of nicked Holliday junctions by RuvC

**Figure 1:**
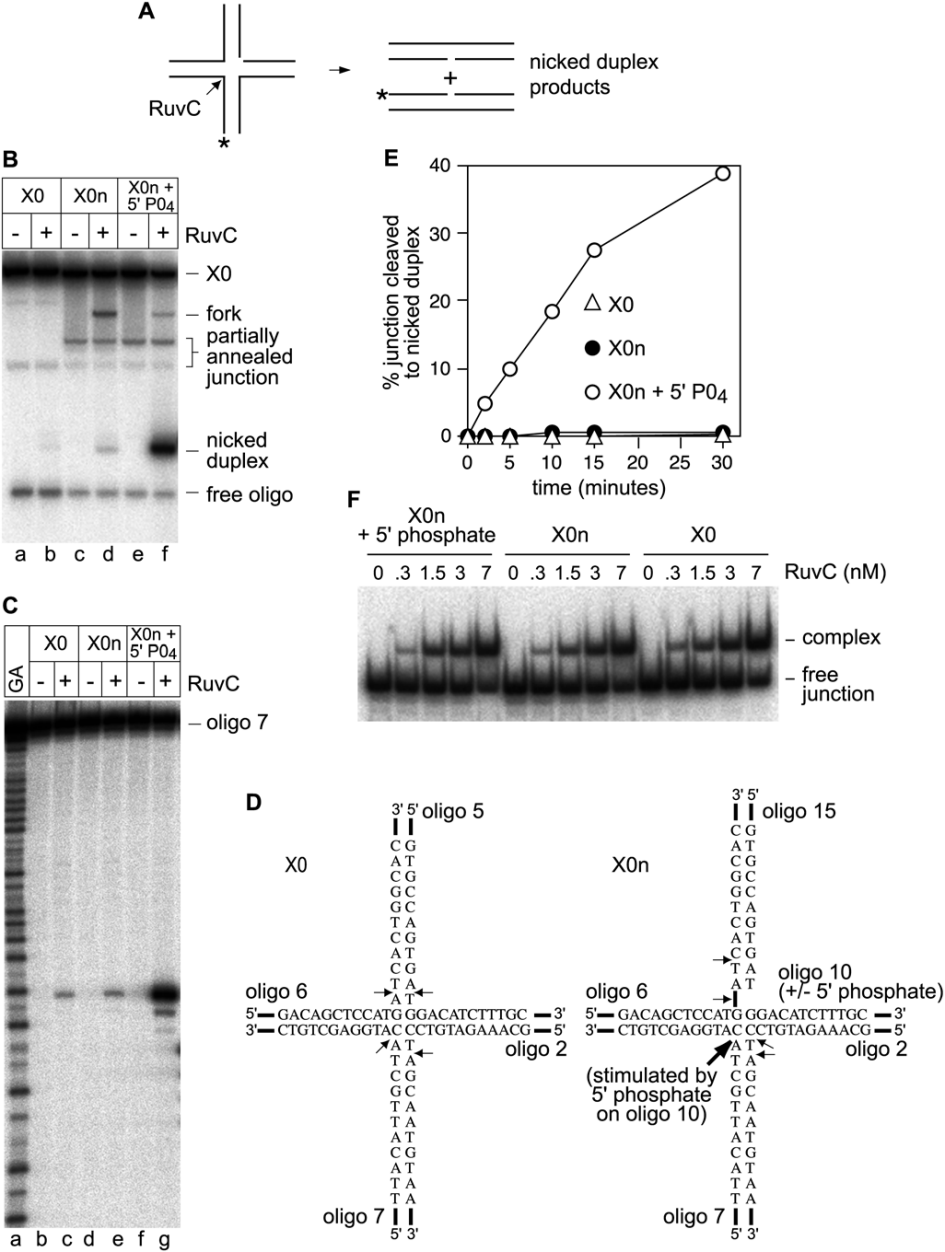
Cleavage of X0 and X0n (+/− 5′ phosphate at nick site) by RuvC. (A) Schematic showing the linear duplex products that are generated by the cleavage of X0 or X0n by RuvC. The asterisk indicates the 5′ ^32^P label. (B) Native polyacrylamide gel showing the cleavage of X0 and X0n (+/− 5′ phosphate at nick site) by RuvC. Reactions (40 µl) contained 1.3 nM junction DNA and 50 nM RuvC as indicated. Reactions were incubated at 30°C for 30 min before being stopped. (C) Denaturing gel of the same reactions as in A. (D) Schematic showing the core nucleotide sequences in X0 and X0n and the sites of cleavage by RuvC. (E) A comparison of the rates of cleavage of X0 and X0n (+/− 5′ phosphate at nick site) by RuvC. Reactions (70 µl) contained 1.4 nM junction DNA and 10 nM RuvC. Data are the mean of three experiments. (F) A comparison of RuvC's binding affinity for X0 and X0n (+/− 5′ phosphate at nick site) by RuvC. Reaction conditions are described in Materials and Methods.

It has been shown previously that a pre-existing strand break at the point of strand exchange within a synthetic HJ stimulates its rate of cleavage by RuvC by 8-fold compared to the corresponding intact junction [6]. However, it has not been determined whether a 5′ phosphate at the strand break is necessary for cleavage stimulation. To investigate this we used intact and nicked versions of a static X-junction (X0 and X0n, respectively), whose point of strand exchange is fixed by sequence heterology between its four junction arms. For optimal cleavage RuvC requires that the consensus sequence 5′-^A^/_T_TT^G^/_C_-3′ is present in opposite strands symmetrically positioned at the point of strand exchange [4]. X0 does not contain this consensus in any of its strands at the strand exchange point (Figure 1D). Nevertheless, RuvC can weakly cleave X0 to generate nicked duplex products (Figure 1A, and 1B, lane b). To map the cleavage sites in X0 four identical junctions were made, each of which was 5′ end-labelled in a different strand. Following incubation with RuvC the reaction products were run on a denaturing gel adjacent to appropriate sequencing ladders (Figure 1C and Supplementary Figure S1). A single main cleavage site was detected in each of the four junction strands at or within one nucleotide of the junction crossover point (Figure 1C, lane c, 1D, and Supplementary Figure S1, lanes c, h and m). To see what effect a pre-existing nick at the point of strand exchange has on RuvC's ability to cleave X0, we used X0n, which contains a pre-existing nick that is exactly symmetrical with the RuvC cleavage site in oligonucleotide 7 of X0 (Figure 1D). Without a 5′ phosphate at the nick site RuvC resolves X0n into nicked duplex products at a similar rate as X0 (Figure 1E), and the position it cleaves in oligonucleotide 7, which is opposite the nick, is unaffected (Figure 1C, compare lanes c and e). RuvC is still able to cleave X0n in oligonucleotides 2 and 6, albeit in slightly different positions than in X0, resulting in a fork product (Figure 1B, lane d, 1D, and Supplementary Figure S1, lanes d and i). The presence of a 5′ phosphate at the nick site makes little difference to RuvC's cleavage of oligonucleotides 2 and 6 (Supplementary Figure S1, lanes e and j). However, it makes a big difference to the amount of cleavage opposite the nick in oligonucleotide 7 (Figure 1C, compare lanes e and g). This can also be seen by the increase in nicked duplex product on a native gel (Figure 1B, lane f). A comparison of the rate of cleavage of X0n with and without a 5′ phosphate shows that the presence of a 5′ phosphate increases the cleavage rate by >50-fold (Figure 1E). This improvement in junction cleavage does not correlate with an increase in binding affinity, since X0, X0n (no 5′ phosphate) and X0n (+ 5′ phosphate) are bound equally well by RuvC (Figure 1F).

**Figure 2:**
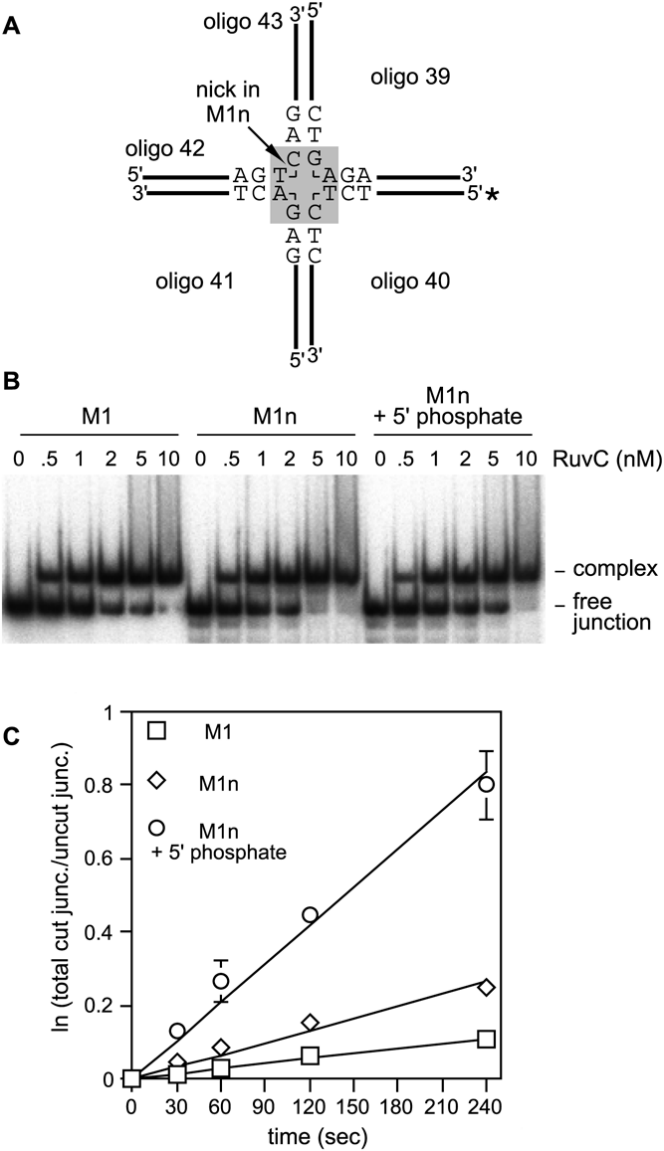
Cleavage of M1 and M1n (+/− 5′ phosphate at nick site) by RuvC. (A) Schematic showing the core nucleotide sequences in M1 and M1n. The asterisk indicates the 5′ ^32^P label. (B) A comparison of RuvC's binding affinity for M1 and M1n (+/− 5′ phosphate at nick site) by RuvC. Reaction conditions are described in Materials and Methods. (C) Single turnover kinetic analysis of M1 and M1n (+/− 5′ phosphate at the nick site) cleavage by RuvC. The reaction conditions are described in Materials and Methods. The data are the means of three independent experiments, and the error bars represent the standard deviations.

To confirm that our results were not specific to X0n we performed a similar set of experiments using a different junction called M1 [15], [19]. M1 contains a much better cleavage site for RuvC than X0, albeit it is still sub-optimal (Figure 2A). Derivatives of M1 containing a single-strand break at the RuvC cleavage site opposite oligo 39 were constructed with and without a 5′ phosphate at the break site. These M1n junctions were then compared together with M1 for binding by RuvC (Figure 2B). Similar to X0 and X0n, M1 and M1n (+/− 5′ phosphate) are bound equally well by RuvC. However, under single turnover conditions the presence of a 5′ phosphate at the strand break in M1n results in a marked stimulation of cleavage rate by RuvC, with a first-order rate constant of 0.209 min^−1^ compared to 0.067 min^−1^ for M1n and 0.028 min^−1^ for M1. These data, together with those obtained using X0 and X0n, indicate that the acceleration of the second strand cleavage during the resolution of a HJ by RuvC depends to a large extent on the 5′ phosphate that is exposed by cleavage of the first strand.

### A 5′ phosphate is not needed by Mus81 for optimal cleavage of nicked HJs

**Figure 3:**
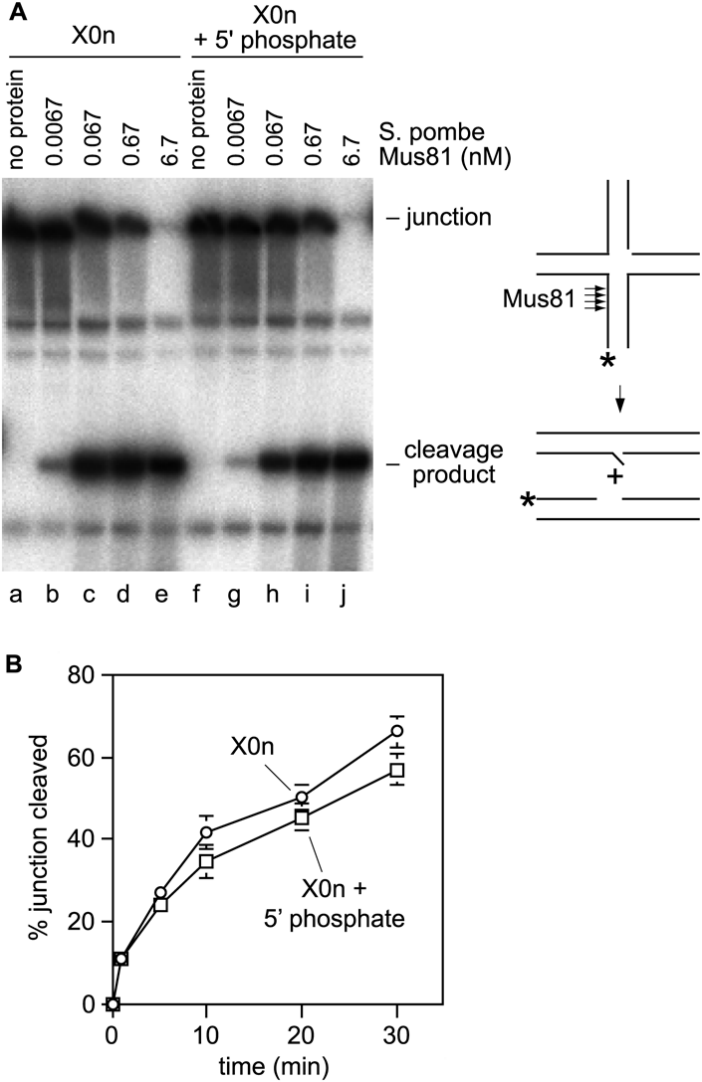
A comparison of the cleavage of X0n (no 5′ phosphate at nick site) and X0n (+ 5′ phosphate at nick site) by *S. pombe* Mus81-Eme1. (A) Reactions (20 µl) contained 1.1 nM junction DNA and the indicated amounts of protein, and were incubated at 30°C for 30 minutes before being stopped and run on a 10% native polyacrylamide gel. The schematic on the right-hand side of the panel shows the duplex products that are generated by the cleavage of X0n by Mus81. The asterisk indicates the 5′ ^32^P label. (B) Time courses of X0n (+/− 5′ phosphate at the nick site) cleavage by Mus81. Reactions (40 µl) contained 2 nM junction DNA and 0.2 nM Mus81-Eme1. Values are means±standard error of the mean from three independent experiments.

It was conceivable that a 5′ phosphate might be necessary to promote the flexibility of a nicked HJ, and therefore would generally enhance cleavage by enzymes that work poorly on “rigid” junctions. An example of such an enzyme is Mus81-Eme1, which is believed to favour binding and cleavage of nicked HJs over fully ligated HJs due to the flexibility generated by the strand discontinuity [18]. We have previously shown that recombinant *Schizosaccharomyces pombe* Mus81-Eme1 (referred to as Mus81 hereafter) readily cleaves X0n to produce a mixture of duplex products containing a single-strand gap or 5′ flap [17]. However, the version of X0n that was used in these experiments did not contain a 5′ phosphate at the nick site. We therefore tested whether Mus81's ability to cleave X0n is enhanced by the addition of a 5′ phosphate at the nick site (Figure 3A). In contrast to RuvC, the presence of a 5′ phosphate at the nick site makes little or no difference to Mus81's ability to cleave X0n (compare lanes b–e with g–j). To confirm this result we also monitored the cleavage of X0n plus and minus a 5′ phosphate in a time course experiment (Figure 3B). Again no significant difference was observed in the rate of cleavage of X0n with and without a 5′ phosphate. These data suggest that nicked X-junctions with and without a 5′ phosphate do not differ dramatically in their flexibility, at least in terms of that required to promote efficient cleavage by Mus81.

### Mus81 cleavage site selection is affected by a 5′ phosphate

**Figure 4:**
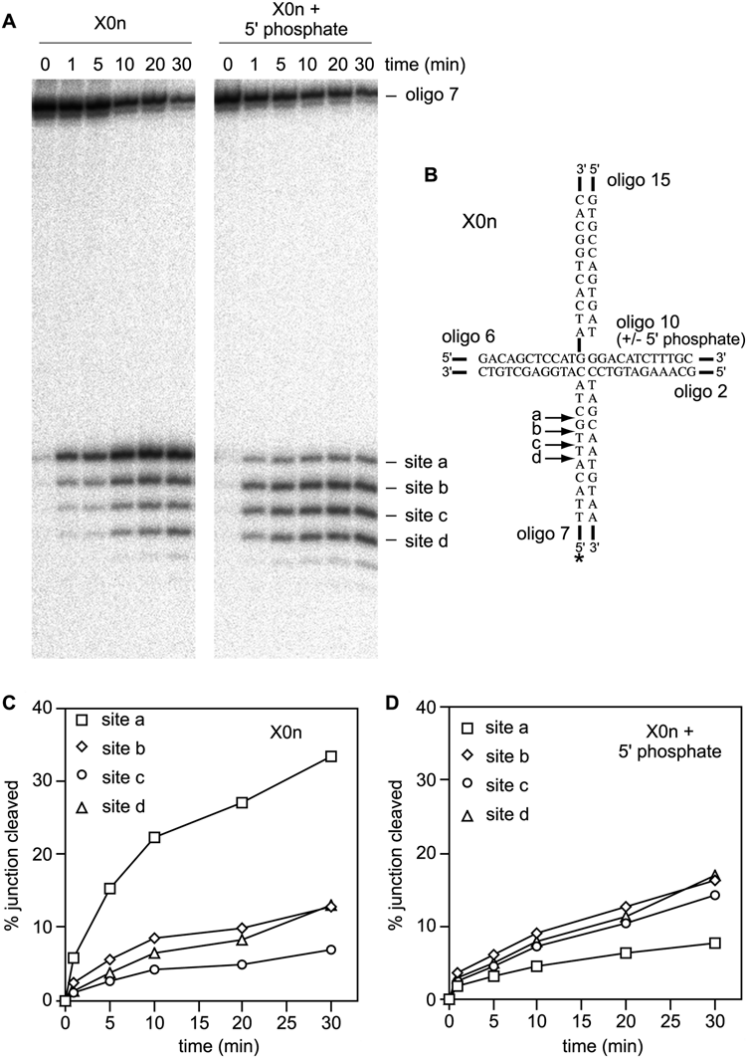
Effect of a 5′ phosphate at the nick site in X0n on cleavage site preference by Mus81-Eme1. (A) Denaturing gels showing time courses of cleavage at sites a–d in X0n (+/− 5′ phosphate at the nick site) by Mus81. Reactions (40 µl) contained 2 nM junction DNA and 0.2 nM Mus81-Eme1. (B) Schematic showing the core nucleotide sequences in X0n and the main sites of cleavage by Mus81. (C and D) Mean data from three experiments like shown in A. Error bars are omitted for the sake of clarity.

Although a 5′ phosphate has no effect on the efficiency of X0n cleavage by Mus81, it might affect the position of the cleavage site. Indeed we have shown previously that the position of the 5′ DNA end in relation to the junction crossover point plays an important role in directing the site of cleavage [17]. To see if a 5′ phosphate affects cleavage site selection by Mus81 we analysed the products of X0n (+ and − 5′ phosphate) cleavage reactions on denaturing gels (Figure 4A). As shown previously Mus81 cleaves X0n at four main sites (a–d) 5′ to the junction crossover point in the strand that is symmetrical to the nick, with a strong preference for site A [17] (Figure 4A and B). The addition of a 5′ phosphate does not change the position of these cleavage sites, but does significantly alter cleavage site preference - the preferred cleavage sites are shifted further away from the point of strand exchange, with increased levels of cleavage at sites b, c and d (Figure 4A and B).

Mus81 can cleave nicked and gapped DNA duplexes [14], [20] therefore it is possible that some of the cleavage sites detected in X0n may result from secondary cleavage events. Indeed it has been proposed that cleavages at sites b, c and d in X0n result from Mus81 acting on the gapped duplex generated from site a cleavage [14]. Therefore the 5′ phosphate at the nick site may mediate its effect on cleavage site position at the level of the gapped duplex rather than the nicked X-junction. To investigate this we monitored the rate of cleavage at sites a–d on X0n by Mus81 (Figure 4A, C and D). Without a 5′ phosphate at the nick site X0n is cleaved fastest at site a, whereas sites b–d are cleaved at much slower rates. These data are consistent with the idea that cleavage at sites b–d result from secondary events [14]. However, X0n with a 5′ phosphate at the nick site is cleaved fastest at sites b, c and d, which is not indicative of secondary events. These data indicate that a 5′ phosphate at the nick site in X0n directs Mus81 to preferentially cleave at sites b, c and d rather than at site a.

## Discussion

Many HJ resolvases, including RuvC, cleave HJs by two consecutive, but uncoupled, strand cleavages [1]. To ensure that bilateral strand cleavage is achieved within the lifetime of a single resolvase-HJ complex, incision of the second strand is accelerated compared to that of the first strand. One possible explanation for this is that cleavage of the first strand is slowed by the need to distort the junction in order to position the scissile bond within the active site [1]. However, once the first strand is cleaved, the junction becomes more flexible, and therefore the second scissile bond can be more readily located into the resolvase's active site. One of the supporting pieces of evidence for this is that the rate of strand cleavage in a nicked junction, containing a consensus RuvC cleavage site, is 8-fold higher than in the same junction without a nick [6]. Our own data shows that the rate of cleavage of M1n is ∼2.4 fold higher than M1, which is consistent with the idea that increased junction flexibility aids second strand cleavage. However, the rate of cleavage is increased by a further ∼3.1 fold if the nick contains a 5′ phosphate. In the case of X0, which contains a very poor RuvC cleavage site, the effect is even more dramatic with the 5′ phosphate stimulating cleavage by more than 50 fold compared to the same junction without a 5′ phosphate. These data suggest that the acceleration of second strand cleavage during the resolution of a HJ by RuvC depends to a large extent on the exposure of a 5′ phosphate.

Why is the 5′ phosphate critical for accelerating second strand cleavage by RuvC? One possibility is that a nicked HJ without a 5′ phosphate is not as flexible as one with a 5′ phosphate. We are unaware of any study that has directly addressed this possibility, however it has been reported that the phosphates at the centre of an intact HJ influence junction conformation [21]. However, we think that the presence of a 5′ phosphate is unlikely to have any major effect on the flexibility of a nicked HJ. Certainly it does not improve the efficiency of X0n cleavage by Mus81, which is thought to require considerable junction flexibility for proper complex formation [18].

A second possibility is that the 5′ phosphate provides a molecular “handle” for RuvC to interact with thereby enabling it to influence junction conformation in a way that enhances second strand cleavage. This would be analogous to another member of the RNase H/Integrase superfamily, Tn5 transposase, which interacts with the 5′ phosphate exposed by hairpin cleavage during the transposition reaction [22]. The coordination of the 5′ phosphate involves residues of the (R)YREK motif that is common to the IS*4* transposase family, and stabilizes a DNA conformation that dramatically enhances strand transfer of the donor DNA into the target by promoting target DNA capture and/or the strand transfer reaction itself [22].

A third possibility is that the putative interaction between RuvC and the 5′ phosphate generates a conformational change in RuvC itself that, together with the additional flexibility of the nicked HJ, stimulates second strand cleavage. Here we imagine that charge repulsion or attraction between the exposed phosphate and residue(s) in the active site of the first monomer might help to promote a conformational change, which could in some way be relayed to the active site of the second monomer aiding its interaction with the scissile bond. Indeed the idea that a conformational change in one subunit can be relayed to a second subunit has been mooted to explain the enhancement of second strand cleavage by the HJ resolvase Ydc2 [23]. Structural studies of RuvC and its interaction with nicked HJs with and without a 5′ phosphate will be needed to determine whether or not the phosphate promotes protein and/or DNA conformational changes that can account for the dramatic stimulation of second strand cleavage during junction resolution.

In contrast to RuvC, Mus81 does not need a 5′ phosphate at the nick site to stimulate its ability to cleave nicked HJs. The presence of the nick itself regardless of its terminal chemistry seems to be sufficient for optimal cleavage efficiency. Recently we showed that nicked HJs are bound with higher affinity than intact HJs in the presence of a relatively low concentration of divalent metal ion [15]. Similar to cleavage efficiency, the binding affinity of Mus81 for nicked HJs is unaffected by the presence of a 5′ phosphate at the nick site. This correlation between binding affinity and cleavage efficiency contrasts with RuvC, which binds equally well to intact and nicked HJs (with and without a 5′ phosphate at the nick site) even though optimal cleavage of a nicked HJ depends on the presence of a 5′ phosphate at the nick site. We suspect that optimal binding and cleavage by Mus81 simply requires a junction with the level of flexibility that is achieved by the presence of a strand nick at or close to the junction crossover point.

Although the presence of a 5′ phosphate at the nick site of a nicked HJ has no effect on the activation of Mus81 cleavage, it does influence cleavage site selection. A recent model of the Mus81-Eme1-nicked HJ complex shows how the exposed 5′ DNA end may be close to residues in and around helix 5 of Mus81 [18]. These residues include a conserved aspartate, and therefore it is possible that charge repulsion could cause movement of the 5′ side of the nick away from helix 5, which in turn would “drag” the cleavage site further from the junction crossover point.

### Conclusion

In this study we have shown that the presence of a 5′ phosphate at the strand discontinuity in a nicked HJ plays an important role in stimulating junction cleavage by RuvC. From this we conclude that the acceleration of second strand cleavage during HJ resolution by RuvC is not solely promoted by increased junction flexibility caused by incision of the first strand as previously proposed [1]. Whether a 5′ phosphate is similarly important for efficient bilateral strand cleavage by other HJ resolvases is yet to be determined. However, our observation that Mus81 cleaves nicked HJs with and without a 5′ phosphate with equal efficiency suggests that at least in some cases a nick may only be needed to impart junction flexibility.

## Materials and Methods

### Proteins

Recombinant *Schizosaccharomyces pombe* Mus81-Eme1 was overexpressed *in E. coli* and purified as described previously [15]. RuvC was overexpressed from plasmid pGS775 in BL21 (DE3) pLysS and purified as described [24] with modifications described in [15]. Protein concentrations were estimated using a protein assay kit (Bio-Rad) with bovine serum albumin as the standard. Amounts of RuvC are expressed in moles of monomer, and Mus81-Eme1 is expressed in moles of dimers of heterodimers.

### DNA substrates

The oligonucleotides used to make X0, X0n, M1 and M1n have been described previously [15], [17]. Oligonucleotides were supplied by Sigma-Genosys Ltd. and were purified by electrophoresis through a 15% (w/v) denaturing gel, full-length bands being cut out and extracted from the gel by soaking in TE (10 mM Tris-HCl, pH 8.0, 1 mM EDTA) overnight. Oligonucleotides were phosphorylated at their 5′-ends where indicated using ATP and polynucleotide kinase. The procedures for annealing and substrate preparation have been described previously [25], [26]. DNA substrates were radiolabelled at the 5′-end of one of their component oligonucleotides as indicated using [γ-^32^P]ATP and polynucleotide kinase. The concentration of DNA substrates was estimated by relating the specific activity of the labelled oligonucleotide to the activity of the purified substrate, and is expressed in molar concentrations of DNA substrate.

### Nuclease assays

Reactions were in 25 mM Tris-HCl, pH 8.0, 1 mM DTT, 100 µg/ml bovine serum albumin, 6% (v/v) glycerol, and contained either 2.5 mM MgCl_2_ (Figures 3 and 4) or 10 mM MgCl_2_ (Figures 1 and 2), as well as the indicated amounts of radiolabelled DNA substrate. The cleavage reactions in Figures 1, 3 and 4 were started by the addition of enzyme and then incubated at 30°C for the indicated amount of time before being stopped by the addition of one-fifth volume of stop mixture (2.5% SDS, 200 mM EDTA, 10 mg/ml proteinase K) followed by a further 15 min at 30°C to deproteinize the mixture. For the single-turnover kinetic analysis of junction cleavage (Figure 2) 1 nM of radiolabelled junction DNA was pre-incubated with 100 nM RuvC in 25 mM Tris-HCl, pH 8.0, 1 mM DTT, 100 µg/ml bovine serum albumin, 6% (v/v) glycerol for 5 minutes at 37°C in a total volume of 40 µl. In preliminary experiments it was established that all of the junction DNA was bound by RuvC under these reaction conditions (data not shown). Cleavage was then initiated by the addition of MgCl_2_ to a final concentration of 10 mM. 8 µl samples were then withdrawn into stop mixture at timed intervals and processed ready for gel electrophoresis as described above. Reaction products were analyzed by electrophoresis through 10% native polyacrylamide gels in Tris borate/EDTA (TBE) buffer at 200 V for 2 h and/or 15% denaturing gels containing 7 M urea. For native gels, deproteinated reactions were mixed with loading dye and loaded directly onto the gel. For denaturing gels, reactions were extracted with phenol/chloroform/isoamyl alcohol (25∶24∶1), and the DNA was precipitated with ethanol, washed twice with 70% ethanol, resuspended in gel-loading buffer (0.05% (w/v) bromophenol blue, 0.05% (w/v) xylene cyanol, 10 mM EDTA, pH 7.5, 97.5% (v/v) formamide), and denatured by boiling for 2 min before loading onto the gel. To map cleavage sites reaction products were run alongside Maxam-Gilbert GA sequence ladders of the appropriate labelled oligonucleotide. A 1.5-base allowance was made to compensate for the nucleoside eliminated in the sequencing reaction. Gels were dried onto 3 MM Whatman paper and analyzed by Phosphor Imaging using a Fuji FLA3000 and Image Gauge V3.3 software. Single turnover rate constants were calculated by measuring the gradient of ln (cut junction/uncut junction) against time in minutes by linear regression.

### Junction binding assays

Reactions (20 µl) contained either 0.6 nM (Figure 1) or 1 nM (Figure 2) radiolabelled junction DNA in 25 mM Tris-HCl, pH 8.0, 1 mM DTT, 100 µg/ml bovine serum albumin, 6% (v/v) glycerol and protein as indicated. The reactions were started by the addition of protein and then incubated at room temperature for 10 minutes before loading onto a 4% native polyacrylamide gel in low ionic strength buffer (6.7 mM Tris-HCl (pH 8.0), 3.3 mM sodium acetate, 2 mM EDTA). The gel and running buffer were pre-cooled at 4°C, and then run at room temperature at 160V for 2 hours with buffer recirculation following sample loading. Gels were dried on 3MM Whatman paper, and then analysed by Phosphor Imaging using a Fuji FLA3000.

## Supporting Information

Figure S1 Mapping RuvC cleavage sites in X0 and X0n (+/− 5′ phosphate at nick site). Denaturing gel showing the RuvC cleavage sites in the component oligonucleotides of X0 and Xn (+/− 5′ phosphate at the nick site). Reaction conditions were the same as described for Figure 1B.
(1.61 MB TIF)
