# Supplementary figures and images for: Efficient Second Strand Cleavage during Holliday Junction Resolution by RuvC Requires Both Increased Junction Flexibility and an Exposed 5′ Phosphate
Source: PLoS One. 2009 Apr 28;4(4):e5347. doi: 10.1371/journal.pone.0005347 (PMC2670506; doi:10.1371/journal.pone.0005347)

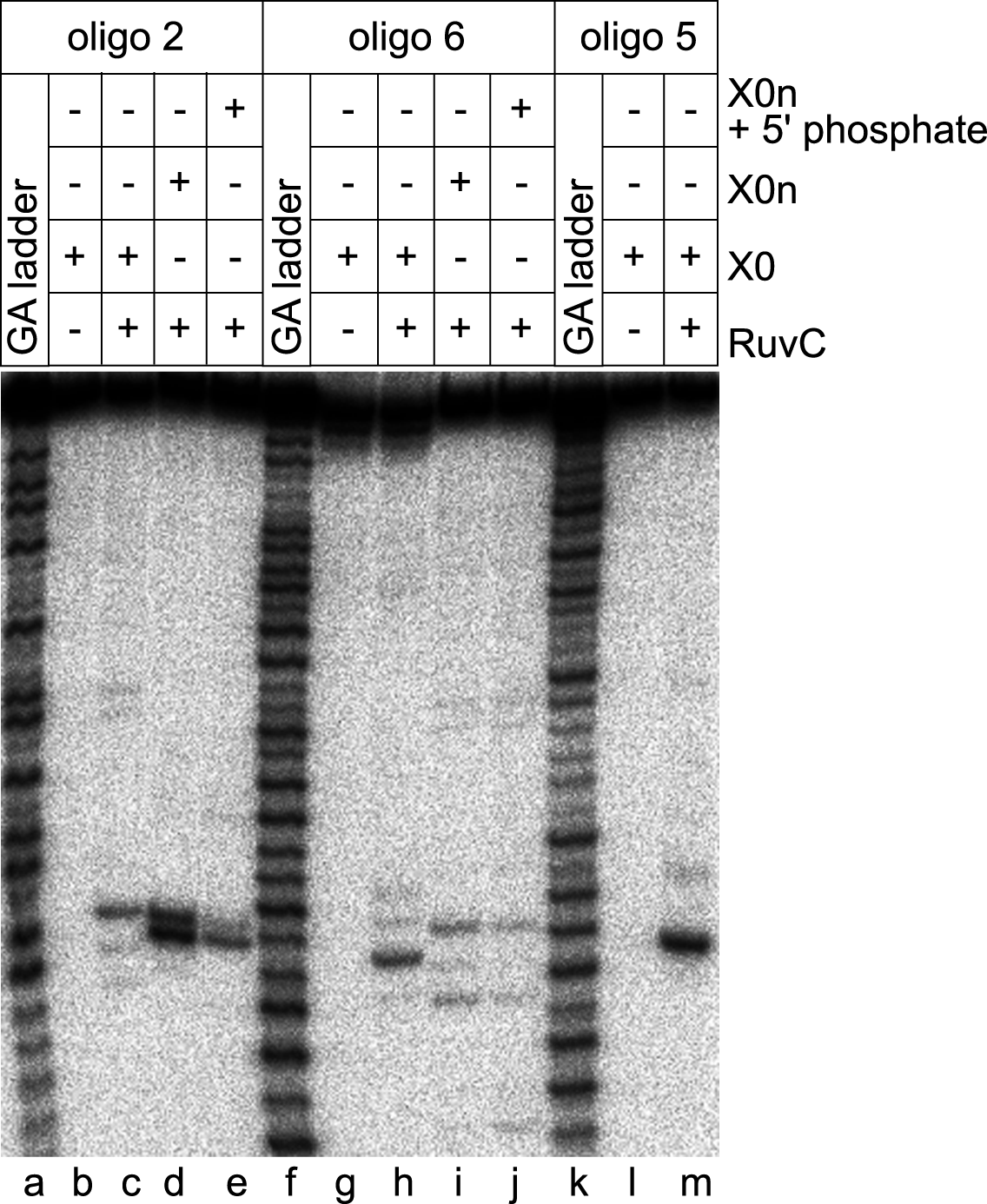

Supplement: Figure S1 — Mapping RuvC cleavage sites in X0 and X0n (+/− 5′ phosphate at nick site). Denaturing gel showing the RuvC cleavage sites in the component oligonucleotides of X0 and Xn (+/− 5′ phosphate at the nick site). Reaction conditions were the same as described for Figure 1B. (1.61 MB TIF) [file pone.0005347.s001.tif]
